# Trends in Lipids and Lipoproteins Among Adults in Northwestern Xinjiang, China, From 1998 Through 2015

**DOI:** 10.2188/jea.JE20180018

**Published:** 2019-07-05

**Authors:** Ling Zhou, Xin Zhao, Mulalibieke Heizhati, Suofeiya Abulikemu, Delian Zhang, Qiuyan Cheng, Weijin Ouyang, Xiaoguang Yao, Jing Hong, Ting Wu, Zuhere Xiamili, Nanfang Li

**Affiliations:** The Center of Hypertension of the People’s Hospital of Xinjiang Uygur Autonomous Region, The Hypertension Institute of Xinjiang, Xinjiang, China

**Keywords:** lipid, dyslipidemia, ethnicity, epidemiologic study, risk factors

## Abstract

**Background:**

To examine trends in serum lipids in population in Northwestern Xinjiang between 1998 and 2015 and to provide clues for future prevention.

**Methods:**

We enrolled 5,142 adults aged ≥30 years from seven independent cross-sectional studies conducted in 1998–2000, 2007–2008, and 2015. Blood lipid profiles, such as total cholesterol (TC), triglyceride (TG), high-density lipoprotein cholesterol (HDL-C), and low-density lipoprotein cholesterol (LDL-C), were measured.

**Results:**

The mean age was 48.5 years in 1998–2000, 47.9 years in 2007–2008, and 53.7 years in 2015. There was a declining trend in the prevalence of dyslipidemia among adults in northwestern Xinjiang. Mean LDL-C decreased during the same period, while mean HDL-C showed the opposite trend. Mean TC was 4.79 mmol/L in 1998–2000, 5.17 mmol/L in 2007–2008, and 4.59 mmol/L in 2015. The trend of mean TG was similar to that of TC. The prevalence of dyslipidemia was closely related with male gender, Mongolian ethnicity, hypertension, obesity, elevated fasting blood glucose, smoking, and drinking.

**Conclusion:**

Between 1998 and 2015, favorable trends in lipid levels have occurred among adults of Northwestern Xinjiang. However, further efforts are needed.

## INTRODUCTION

Cardiovascular disease (CVD) is the leading cause of death worldwide and accounts for 31% of all global deaths.^1^ The World Health Organization (WHO) reported that, about 17.7 million people died from CVD worldwide in 2015.^1^ Epidemiologic surveys have demonstrated that high concentrations of total cholesterol (TC) and low-density lipoprotein cholesterol (LDL-C) and low levels of high-density lipoprotein cholesterol (HDL-C) are major risk factors for CVD.^2^^–^^8^ Many previous studies have proved that mortality and morbidity from CVD can be reduced by lowering the serum lipids levels.^3^^,^^4^^,^^9^^–^^12^

Over 28 years, mean TC has decreased from high levels in the high-income western countries, whereas it increased in east and southeast Asia and Pacific by 0.08 mmol/L per decade in males and 0.09 mmol/L per decade in females.^13^ In China, the prevalence of dyslipidemia in adults has increased from 18.6% in 2002 to 40.1% in 2012.^14^^,^^15^ Elevated serum cholesterol levels are expected to result in 9.2 million extra CVD events in China during the next 20 years.^16^

Xinjiang, a vast and multi-ethnic province, is located in the northwest edge of China. It is the largest Chinese administrative region and takes up about one-sixth of the country’s territory. Their major employment is peasants or herdsmen in this agricultural and pastoral area. Previous studies have shown that the prevalence of dyslipidemia in Xinjiang is higher than the national level.^17^^–^^20^ The prevalence of dyslipidemia in Xinjiang was 49.2% in 1997–1998.^19^

This study observed that significant differences also existed in lipid levels and prevalence of dyslipidemia among Kazakh (53.5%), Mongolian (49.3%), and Han (47.3%) ethnic groups, which is likely to be caused by different lifestyle and diet-related habits. Earlier studies have indicated that Kazakhs consume more animal meat and fewer vegetables.^21^ Historically, there was a drastic difference in the living environment between the ethnic groups. Most Kazakhs and Mongolians reside in stock-raising areas like villages, forests, and mountains and move around with seasonal changes, which makes their living depend much on animal products, including animal oil, meat, milk, and dairy products and makes it difficult to have enough intake of vegetables and fruits. Furthermore, some roles of genetics have been described,^22^ although not confirmed. Meanwhile, Xinjiang, previously had lower education attainment (illiteracy rate: about 11.44%, higher than the national level of 9.08% in 2000),^23^ relatively backward economy, and extremely immature medical conditions, which makes it difficult to implement health education. Nonetheless, in recent years, socio-economic conditions, education attainment, and health care resources have shown significant development in Xinjiang. The Gross domestic product increased from 111.66 billion RMB in 1998 to 932.48 billion RMB in 2015.^24^ The proportion of population acquiring senior middle or higher education increased from 19.0% (*n* = 3,194,374) in 2000 to 24.3% (*n* = 4,860,941) in 2010.^23^^,^^25^ Moreover, increases in coverage and utilization of health care resources, particularly the implementation of new rural medical cooperative care system, were seen in China since 2003.^26^ Therefore, it is reasonable to believe that all the changes over these years might have had beneficial effects on lipid levels and on the prevalence of dyslipidemia. However, there have been no subsequent updates of the prevalence of dyslipidemia and trends in lipid profiles in this area.

Therefore, this study aimed to examine recent trends in lipid profiles, including lipid ratios and the prevalence of dyslipidemia, in three counties of northwestern Xinjiang Province based on seven independent population surveys performed in Hefeng (1998, 2008, and 2015), Fuhai (2000 and 2015), and Fukang (2007 and 2015).

## METHODS

### Study population

This study was conducted in accordance with the Declaration of Helsinki and with approval from the Ethics Committee of the People’s Hospital of Xinjiang Uygur Autonomous Region. Results were based on data from seven independent cross-sectional surveys performed in Hefeng (1998, 2008, and 2015), Fuhai (2000 and 2015), and Fukang (2007 and 2015). Methods were similar for all seven surveys: at least 2 days prior to the survey, all the inhabitants ≥30 years of age in these specific area were informed of the survey contents, such as necessity of fasting overnight and physical examination. After signing the informed consent, subjects were enrolled in the study. While analyzing, the data collected in 1998 and 2000 were grouped as 1998–2000, the data collected in 2007 and 2008 as 2007–2008, and data collected in 2015 as the 2015 group. Of the 1,609 people who participated in 1998–2000, complete data were obtained from 1,551 subjects (96.4%). Likewise, 2,331 (96.6%) of 2,412 subjects in 2007–2008 and 2,262 (93.3%) of 2,423 subjects in 2015 participated in the survey. We then excluded subjects with any missing data on lipid profiles and subjects who did not fast for at least 12 h before blood sampling. Finally, data on 1,059 subjects from 1998–2000, 2,144 subjects from 2007–2008, and 1,939 subjects from 2015 were analyzed.

### Data collection

Data was collected during a single clinic visit with doctors or trained nurses using a standard questionnaire in face-to-face interviews. The questions assessed: demographic characteristics (age, sex, ethnicity and address), socioeconomic data (marital status and occupation), lifestyle risk factors (smoking and drinking status), and past medical history. Physical examination was conducted, including measurement of blood pressure, body height, and body weight. Blood pressure was presented as the mean of three measurements using a mercury sphygmomanometer via a standardized procedures recommended by the American Heart Association.^27^ All participants were advised to avoid cigarette smoking, alcohol, caffeinated beverages, tea, and exercise for at least 30 min prior to measurement. Three blood pressure measurements were taken after a rest of at least 5 min, from the unclothed right arm of the person in a sitting position at intervals of at least 1 min. Body weight, height, and WC were measured using standard methods.^28^ Height and Weight were measured to the nearest 0.1 cm and 0.1 kg, respectively, with the participants in light-weight clothing and without shoes. Waist circumference (WC) was measured at the midpoint between the lower rib and upper margin of the iliac crest to the nearest 0.1 cm at the end of a normal expiration. Body mass index (BMI) was calculated by dividing weight by height squared (kg/m^2^).

### Laboratory measurements

Venous blood was drawn from all participants who had fasted for ≥12 h. Blood samples were obtained from median cubital vein into Vacutainer tubes without additives. Serum was separated immediately and stored at −80°C. All blood samples were measured within 1 month in the People’s Hospital of Xinjiang Uygur Autonomous Region. TC, triglyceride (TG), HDL-C, LDL-C, and fasting blood glucose (FBG) were measured using enzymatic methods. Non-HDL-C was calculated as serum TC level minus HDL-C level.

### Diagnostic criteria

According to the prevention standard proposed of dyslipidemia in China in 2016,^29^ TC ≥6.2 mmol/L (240 mg/dL), TG ≥2.3 mmol/L (200 mg/dL), HDL-C <1.0 mmol/L (40 mg/dL), and LDL-C ≥4.2 mmol/L (160 mg/dL) were indicative of high TC, high TG, low HDL-C, and high LDL-C, respectively. Dyslipidemia was defined as high TC, and/or high TG, and/or low HDL-C, and/or high LDL-C, and/or having received during treatment for dyslipidemia in the last 2 weeks. Hypertension was defined as having a systolic blood pressure ≥140 mm Hg, and/or diastolic blood pressure ≥90 mm Hg, and/or receiving anti-hypertensive medication.^30^ Overweight and obesity were defined according to the WHO classifications.^31^ BMI ≥25 kg/m^2^ and BMI ≥30 kg/m^2^ indicated overweight and obesity, respectively. Abdominal obesity was defined as having a WC ≥102 cm for men and ≥88 cm for women.^31^ Smokers were defined as those who smoked at least one cigarette per day and had lasted for at least 6 months. Drinkers were defined as those who drank at least twice per month (more than 640 mL beer or 100 mL Chinese liquor; about 57 g alcohol), and had lasted for at least 6 months.

### Statistical analysis

The database was constructed with Excel 2007 Software (Microsoft Corp, Redmond, WA, USA) by two data managers and was corrected to guarantee the accuracy and integration of the data. The data were analyzed using SPSS 19.0 software (SPSS Inc., Chicago, IL, USA). Continuous variables were presented as means (standard deviations [SDs]) and were analyzed using *t*-test or ANOVA. Categorical variables were expressed as proportion (%) and frequency (*n*) and were analyzed using the Chi-square test. In this analysis, the standardized population was determined using the total study population between 1998 and 2015. Age-standardized prevalence of dyslipidemia for each study period (1998–2000, 2007–2008, and 2015) was then calculated according to the standardized population. As for dyslipidemia, adjusted odds ratios (ORs) with associated 95% confidence intervals (CIs) were calculated using multivariate logistic regression model through the Enter method. All the study population between 1998 and 2015 was included in multivariate logistic regression model. Covariates were gender, age, ethnicity, smoking, drinking, BMI, WC, hypertension, and FBG in the models. All statistical tests were two-sided and *P*-values <0.05 were considered significant.

## RESULTS

### Population characteristics

Table 1 details the characteristics of the study participants. The proportion of male was 41.5% in 1998–2000, 40.8% in 2007–2008, and 45.5% in 2015. Mean age of the subjects was 48.5 (SD, 11.6) years, 47.9 (SD, 10.9) years, and 53.7 (SD, 13.3) years, respectively. There were differences in ethnic composition among the three groups. The proportion of smokers declined across the survey years, whereas drinking status showed no difference between the survey years. Mean BMI was significantly higher in 2007–2008 populations than in 1998–2000 and 2015 populations, but there were no significant differences observed in the mean level of WC and FBG.

**Table 1.** Demographic characteristics of subjects, 1998–2015

Parameters	1998–2000 (*n* = 1,059)	2007–2008 (*n* = 2,144)	2015 (*n* = 1,939)	*P*
Gender				
Male, *n* (%)	439 (41.5)	874 (40.8)	882 (45.5)	0.006
Female, *n* (%)	620 (58.5)	1270 (59.2)	1057 (54.5)	
Age, years	48.5 (11.6)	47.9 (10.9)	53.7 (13.3)	<0.001
30–39, *n* (%)	243 (22.9)	540 (25.2)	282 (14.5)	
40–49, *n* (%)	361 (34.1)	730 (34.0)	594 (30.6)	
50–59, *n* (%)	251 (23.7)	528 (24.6)	440 (22.7)	
≥60, *n* (%)	204 (19.3)	346 (16.2)	623 (32.2)	
Ethnicity				
Han, *n* (%)	63 (5.9)	454 (21.2)	1158 (59.7)	
Mongolian, *n* (%)	318 (30.0)	496 (23.1)	386 (19.9)	<0.001
Kazakh, *n* (%)	678 (64.1)	1194 (55.7)	395 (20.4)	
Smoking, *n* (%)	267 (25.2)	503 (23.5)	414 (21.4)	0.046
Drinking, *n* (%)	234 (22.1)	462 (21.5)	427 (22.0)	0.912
BMI, kg/m^2^, mean (SD)	25.4 (4.3)	26.7 (4.3)	26.1 (4.2)	<0.001
WC, cm, mean (SD)	—	85.3 (11.4)	90.0 (11.6)	0.806
SBP, mm Hg, mean (SD)	148.3 (31.7)	134.1 (24.9)	128.6 (20.6)	<0.001
DBP, mm Hg, mean (SD)	91.8 (18.1)	86.1 (14.1)	76.4 (11.7)	<0.001
FBG, mmol/L, mean (SD)	—	5.32 (1.24)	5.17 (1.30)	0.481

BMI, body mass index; DBP, diastolic blood pressure; FBG, fasting blood glucose; SBP, systolic blood pressure; SD, standard deviation; WC, waist circumference.

### Trends in lipid profiles

Table 2 shows the trends in lipid profiles. Mean LDL-C decreased from 3.53 (SD, 1.09) mmol/L in 1998–2000 to 2.67 (SD, 0.78) mmol/L in 2015 (*P* < 0.001). Mean TC was 4.79 (SD, 1.15) mmol/L in 1998–2000, 5.17 (SD, 1.04) mmol/L in 2007–2008, and 4.59 (SD, 0.95) mmol/L in 2015. Mean TG was 0.94 (SD, 0.57) mmol/L in 1998–2000, 1.58 (SD, 1.28) mmol/L in 2007–2008, and 1.11 (SD, 0.87) mmol/L in 2015. Mean HDL-C was 1.08 (SD, 0.36) mmol/L in 1998–2000, 1.50 (SD, 0.50) mmol/L in 2007–2008, and 1.44 (SD, 0.33) mmol/L in 2015. Mean non-HDL-C was 3.71 (SD, 1.13) mmol/L in 1998–2000, 3.68 (SD, 1.00) mmol/L in 2007–2008, and 3.16 (SD, 0.90) mmol/L in 2015. As for lipid ratios, the ratios of TC/HDL-C and LDL-C/HDL-C showed decreasing trends from 1998 to 2015 (*P* < 0.001). Mean TG/HDL-C ratios was 0.99 (SD, 0.76) in 1998–2000, 1.20 (SD, 1.06) in 2007–2008, and 0.93 (SD, 0.87) in 2015.

**Table 2.** Serum lipid and lipoprotein levels in subjects, 1998–2015

	1998–2000	2007–2008	2015	*P* value
TC, mmol/L	4.79 (1.15)	5.17 (1.04)	4.59 (0.95)	<0.001
TG, mmol/L	0.94 (0.57)	1.58 (1.28)	1.11 (0.87)	<0.001
HDL-C, mmol/L	1.08 (0.36)	1.50 (0.50)	1.44 (0.33)	<0.001
LDL-C, mmol/L	3.53 (1.09)	2.87 (1.05)	2.67 (0.78)	<0.001
Non-HDL-C, mmol/L	3.71 (1.13)	3.67 (1.02)	3.15 (0.90)	<0.001
TC/HDL-C	5.00 (3.61)	3.72 (1.21)	3.31 (0.85)	<0.001
TG/HDL-C	1.01 (0.76)	1.20 (1.06)	0.93 (0.87)	<0.001
LDL-C/HDL-C	3.80 (2.06)	2.06 (0.87)	1.93 (0.65)	<0.001

HDL-C, high-density lipoprotein cholesterol; LDL-C, low-density lipoprotein cholesterol; TC, total cholesterol; TG, triglyceride.

Data are presented as mean (standard deviation).

### Trends in the prevalence of dyslipidemia

**Figure 1.**
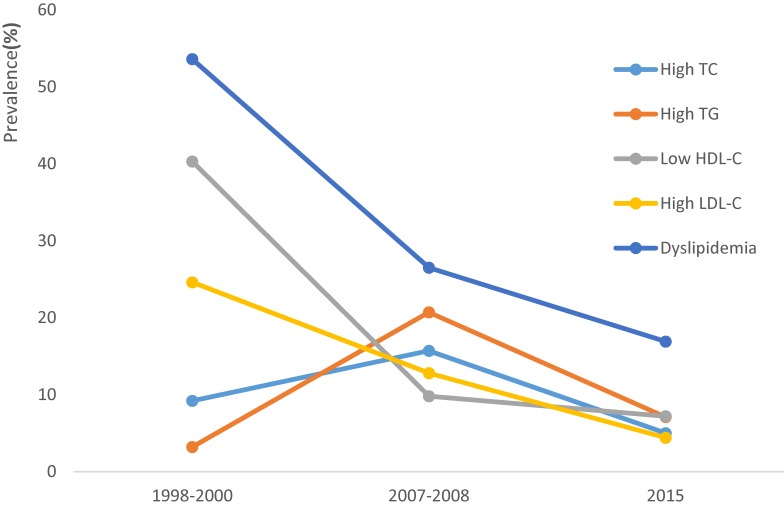
Trends in age-standardized prevalence of high TC, high TG, low LDL-C, high HDL-C, and dyslipidemia from 1998–2015

**Figure 2.**
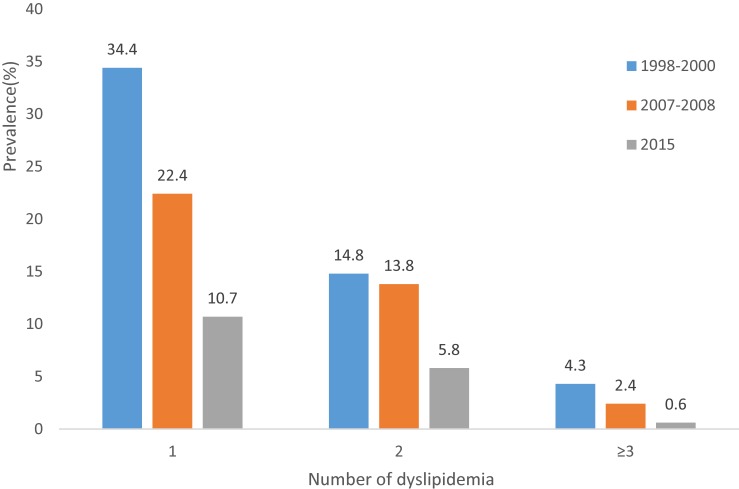
Trends in prevalence of the clustering of dyslipidemia

The trends in the prevalence of dyslipidemia among participants are presented in Table 3. From 1998 to 2015, the prevalence of dyslipidemia decreased from 53.5% in 1998–2000 to 38.6% in 2007–2008 and to 16.8% in 2015. The prevalence of low HDL-C and high LDL-C also showed the same trends across the survey years. Prevalence of high TC was 9.3% in 1998–2000, 14.9% in 2007–2008, and 5.2% in 2015. The prevalence of high TG was 3.2% in 1998–2000, 20.1% in 2007–2008, and 6.9% in 2015, respectively. Figure 1 shows trends in age-standardized prevalence of dyslipidemia. The age-standardized prevalence of dyslipidemia, high TC, high TG, low HDL-C, and high LDL-C was 53.6%, 9.2%, 3.2%, 40.3%, and 24.6% in 1998–2000, 26.5%, 15.7%, 20.7%, 9.8%, and 12.8% in 2007–2008, and 16.9%, 5.0%, 7.1%, 7.2%, and 4.4% in 2015, respectively. The prevalence of the clustering of 1, 2, and ≥3 kinds of dyslipidemia among participants showed significantly declining trends, as seen in Figure 2.

**Table 3.** Trends in prevalence of dyslipidemia, 1998–2015

	1998–2000	2007–2008	2015
High TC	9.3 (7.5–11.0)	14.9 (13.4–16.4)	5.2 (4.3–6.3)
High TG	3.2 (2.1–4.3)	20.1 (18.4–21.8)	6.9 (6.0–8.2)
Low HDL-C	40.4 (37.5–43.7)	10.2 (8.9–11.4)	6.8 (5.8–8.0)
High LDL-C	24.6 (22.0–27.1)	12.2 (10.8–13.6)	4.6 (3.8–5.6)
Dyslipidemia	53.5 (50.5–56.5)	38.6 (36.6–40.6)	16.8 (15.3–18.7)

HDL-C, high-density lipoprotein cholesterol; LDL-C, low-density lipoprotein cholesterol; TC, total cholesterol; TG, triglyceride.

Data are presented as prevalence (95% confidence interval).

### Factors associated with dyslipidemia

As shown in Table 4, multivariate logistic regression analysis suggested that the prevalence of dyslipidemia was closely related with male gender, Mongolian ethnicity, hypertension, obesity, elevated FBG, smoking, and drinking. Table 5 shows the factors associated with high TC, high TG, low HDL-C, and high LDL-C among the participants. The prevalence of high TG, low HDL-C, and high LDL-C were related with BMI (OR 1.574; 95% CI, 1.361–1.822, OR 1.273; 95% CI, 1.071–1.514, and OR 1.523; 95% CI, 1.284–1.807, respectively). Abdominal obesity was a risk factor only for high TG (OR 1.515; 95% CI, 1.158–1.982). Hypertension increased the prevalence of high TC (OR 1.355; 95% CI, 1.091–1.683) and high TG (OR 1.706; 95% CI, 1.403–2.075). The prevalence of high TC, high TG, low HDL-C, and high LDL-C were closely related with FBG (OR 2.362; 95% CI, 1.573–3.545, OR 2.499; 95% CI, 1.740–3.589, OR 2.042; 95% CI, 1.280–3.257, and OR 2.037; 95% CI, 1.299–3.193, respectively). With regard to smoking and alcohol consumption, we observed an association between smoking and the prevalence of high TC, high TG, and high LDL-C (OR 1.666; 95% CI, 1.223–2.269, OR 1.328; 95% CI, 1.019–1.731, and OR 1.489; 95% CI, 1.081–2.051, respectively).

**Table 4.** Factors associated with the prevalence of dyslipidemia from multivariate logistic regression

Variables	Stratification	β	Wald	OR (95% CI)	*P* value
Gender	Female	1			
	Male	0.219	5.304	1.245 (1.033–1.501)	0.021
Age, years	30–39	1			
	40–49	−0.036	0.120	0.965 (0.788–1.182)	0.730
	50–59	0.054	0.244	1.056 (0.081–1.311)	0.622
	≥60	−0.097	0.700	0.907 (0.722–1.140)	0.403
Ethnicity	Han	1			
	Mongolian	0.200	4.149	1.221 (1.008–1.479)	0.042
	Kazakh	−0.171	3.838	0.843 (0.710–1.000)	0.050
Body mass index	Normal	1			
	Overweight	0.423	26.093	1.527 (1.298–1.797)	<0.001
	Obesity	0.653	31.723	1.922 (1.531–2.413)	<0.001
Abdominal obesity	No	1			
	Yes	0.254	5.047	1.289 (1.033–1.609)	0.025
Hypertension	No	1			
	Yes	0.270	12.540	1.310 (1.128–1.522)	<0.001
FBG, mmol/L	<7.0	1			
	≥7.0	0.907	29.228	2.477 (1.783–3.442)	<0.001
Smoking	No	1			
	Yes	0.255	6.145	1.290 (1.055–1.579)	0.013
Drinking	No	1			
	Yes	0.279	6.932	1.322 (1.074–1.628)	0.008

CI, confidence interval; FBG, fasting blood glucose.

**Table 5.** Factors associated with the prevalence of lipid parameters abnormality

Lipid parameters	Factors	β	Wald	OR (95% CI)	*P* value
High TC	Age	0.307	33.057	1.359 (1.224–1.509)	<0.001
	Ethnicity	0.339	28.306	1.403 (1.239–1.590)	<0.001
	Hypertension	0.304	7.561	1.355 (1.091–1.683)	0.006
	Fasting blood glucose	0.859	17.202	2.362 (1.573–3.545)	<0.001
	Smoking	0.511	10.494	1.666 (1.223–2.269)	0.001
High TG	Age	−0.125	6.955	0.883 (0.804–0.968)	0.008
	Ethnicity	−0.475	64.873	0.622 (0.554–0.698)	<0.001
	Hypertension	0.534	28.680	1.706 (1.403–2.075)	<0.001
	Body mass index	0.454	37.198	1.574 (1.361–1.822)	<0.001
	Abdominal obesity	0.415	9.172	1.515 (1.158–1.982)	0.002
	Fasting blood glucose	0.916	24.589	2.499 (1.740–3.589)	<0.001
	Smoking	0.284	4.413	1.328 (1.019–1.731)	0.036
	Drinking	0.347	7.242	1.416 (1.099–1.823)	0.007
Low HDL-C	Gender	0.364	5.903	1.438 (1.073–1.929)	0.015
	Age	−0.260	20.131	0.771 (0.689–0.864)	<0.001
	Body mass index	0.241	7.461	1.273 (1.071–1.514)	0.006
	Fasting blood glucose	0.714	8.977	2.042 (1.280–3.257)	0.003
High LDL-C	Gender	0.309	4.278	1.362 (1.016–1.825)	0.039
	Age	0.235	16.590	1.265 (1.130–1.416)	<0.001
	Ethnicity	0.291	17.723	1.338 (1.168–1.532)	<0.001
	Body mass index	0.421	23.281	1.523 (1.284–1.807)	<0.001
	FBG	0.711	9.607	2.037 (1.299–3.193)	0.002
	Fasting blood glucose	0.398	5.942	1.489 (1.081–2.051)	0.015

HDL-C, high-density lipoprotein cholesterol; LDL-C, low-density lipoprotein cholesterol; TC, total cholesterol; TG, triglyceride.

## DISCUSSION

This is the first report on secular trends in serum lipids in low income northwestern Xinjiang Province, mainly comprised of Han, Kazakh, and Mongolian ethnicities. Our study has found that there was a declining trend in the prevalence of dyslipidemia from 1998–2000 through 2007–2008 and to 2015. There are several reasons for the changes of epidemiological characteristics of dyslipidemia in northwestern Xinjiang. First of all, with the improvement of economic and education level, health awareness has been improved.^23^^–^^25^ Moreover, the Chinese government has increased its investment in medical and health services in recent years.^26^ In addition, since the establishment in 1997, our center has been focusing on the population-based prevention of CVD in Xingjiang by training local health workers. Furthermore, in 2009, the Chinese government launched the construction of nomads settlement, and 110.5 thousand nomadic families were supported by the government, which brought about higher education attainment and health awareness improvement, followed by healthy lifestyles and eating habits, such as more intake of fresh vegetables and fruits. Furthermore, nationwide data suggest that patients with dyslipidemia showed an improvement in lipid lowering treatment and in achieving therapeutic goals in LDL-C over the past decade. For example, 61.5% of very high and high risk patients with lipid-lowering agents in 2012 achieved the therapeutic goal of LDL-C, compared to only 16.6% in 2000.^32^^,^^33^ A similar trend of lipids was found in the developed countries. Declines in mean TC and LDL-C have been recently noted in high-income regions, such as Australasia, North America, and Western Europe.^13^^,^^34^^,^^35^ Although the mean LDL-C was still higher in the United States than in China between 1988 and 2010, the mean TC, TG, LDL-C, and non-HDL-C levels decreased and the mean HDL-C level increased linearly.^36^ Among Chinese adults, the prevalence of dyslipidemia has been increasing during the past decade. Statistics from The National Nutrition and Health Survey in 2002 reported that the prevalence of high TC, high TG, and low HDL-C was 2.9%, 11.9%, and 7.4%, respectively.^14^ In 2012, a survey of adults in China showed that the prevalence of high TC, high TG, and low HDL-C was 4.9%, 13.1%, and 33.9%, respectively.^15^ The increased prevalence of dyslipidemia may be related to dietary pattern changes (such as a higher intake of fat and reduced fiber intake and lower physical activity levels).

We found that the prevalence of dyslipidemia was closely related with male gender, Mongolian ethnicity, hypertension, obesity, elevated FBG, smoking, and drinking in multivariate logistic regression analysis. Compared with females, males had greater odds of having dyslipidemia (OR 1.245), which is in accordance with most previous studies.^37^^,^^38^ However, one study found that dyslipidemia was more prevalent in women.^39^ Our study demonstrated that the prevalence of low HDL-C was higher in men, similar to the result of previous surveys.^40^ In this study, we found that the prevalence of high TC and LDL-C closely related with age. A similar trend of lipid distribution was found in previous studies.^37^^,^^41^ In our study, the risk of dyslipidemia in Mongolian participants was 1.221 times higher than in Han participants. With regard to the differences across ethnic groups in the prevalence of dyslipidemia, the mechanisms are not clear. Most Kazakh and Mongolian live as herders and reside in the pastoral areas north of Xinjiang. In addition, Kazakh and Mongolian share similar dietary habits, characterized by eating more animal fat and consuming less grain.^42^ Kazakh ethnicity is mainly distributed in Kazakhstan, China, Russia, Uzbekistan, Turkey, and Mongolia, while the Mongolians are mainly distributed in China, Mongolia, and Russia. Therefore, the current study may provide a theoretical basis for the prevention and treatment of dyslipidemia in the two ethnicities, which share common lifestyles.

Moreover, other environmental factors, such as cigarette smoking, alcohol consumption, and physical inactivity, are determinants of HDL-C level. Smoking and drinking can decrease the HDL-C levels.^43^ Increased physical activity is known to be related to higher HDL-C level.^44^ The relationship between dyslipidemia and conventional factors, including BMI, WC, BP, and FBG, were also analyzed. Prevalence of high TG, high LDL-C, and low HDL-C was all closely associated with BMI, in agreement with those of previous studies.^37^^,^^44^ Dyslipidemia is more prevalent in abdominal obesity. However, WC was a risk factor only for high TG after adjusting for other factors, possible due to the fact that the participants in this study have less WC and the effect on lipid is corrected by co-existing factors. Hypertension increased the prevalence of high TC and high TG, in accordance with existing evidence.^38^^,^^45^ In addition, all kinds of dyslipidemia were closely associated with FBG.

The present study also has several limitations. First, there were differences in population composition regarding age, gender, and ethnicity among the three groups, which might have brought about biased information. Second, we did not have information available on our participants regarding participation in dietary factors, family income, and other factors that increase the risk of developing dyslipidemia. In addition, important information that affects lipid levels and can explain the lowering prevalence and lipid levels, like use of lipid lowering medicine and physical activity, were not included in the analysis, which made some of conclusions based on speculations. Finally, this study was limited to population from three counties of Northwestern Xinjiang, where a long-term health provider education program of CVD risk factors, especially hypertension, has been implemented since 1997; therefore, the results may not be representative for the whole of Xinjiang Province.

In conclusion, there was a decrease in the prevalence of dyslipidemia among adults of northwestern Xinjiang from 1998 through 2015. In addition, we also examined various risk factors for dyslipidemia. Our results are expected to be helpful in developing appropriate prevention and control strategies for these modifiable risk factors of dyslipidemia and decrease the overall CVD mortality and morbidity.
